# Arterial elementary calcium signaling in aging

**DOI:** 10.18632/aging.104220

**Published:** 2020-12-27

**Authors:** Mario Kassmann, Gang Fan, Maik Gollasch

**Affiliations:** 1 Charité-Universitätsmedizin Berlin, Experimental and Clinical Research Center, a Joint Cooperation between the Charité Medical Faculty and the Max Delbrück Center for Molecular Medicine, Berlin, 13125, Germany; 2 Department of Internal Medicine and Geriatrics, University Medicine Greifswald, Greifswald, Germany, Greifswald, 17489, Germany; 3 Department of Urology, Huazhong University of Science and Technology Union Shenzhen Hospital, The 6th Affiliated Hospital of Shenzhen University Health Science Center, Shenzhen, China; 4 Charité-Universitätsmedizin Berlin, Medical Clinic for Nephrology and Internal Intensive Care, Campus Virchow, Berlin, 13353, Germany

**Keywords:** aging, calcium sparks, caveolae, ryanodine receptors, T-type calcium channels, vascular smooth muscle

Arterial blood pressure increases during aging [1]. Peripheral arteries and arterioles contribute to the increased systemic vascular resistance in hypertension. Resistance sized arteries adapt to hemodynamic forces through changes in their diameter. Myogenic tone is the ability of the vascular smooth muscle cells (VSMC) in the blood vessel wall to constrict when the vessel is radially stretched to cause blood flow autoregulation [2]. A number of artery-related changes contributing to age-dependent increases in blood pressure have been identified: increased vessel stiffness, vascular calcification, alterations by age-associated inflammatory processes, structural changes/vascular remodeling, e.g. increased passive media cross-sectional area [3,4].

Others and we have focused on mechanisms controlling VSMC contraction/relaxation states to regulate vessel diameter. An indirect mechanism involving elementary Ca^2+^ release events (Ca^2+^-sparks) has been identified to limit myogenic tone. This functional unit involves type 2 ryanodine receptors (RyR2s) [2], large-conductance Ca^2+^-sensitive K^+^ (BK_Ca_) channels, causing spontaneous transient outward K^+^ currents (STOCs), Ca_V_1.2 L-type Ca^2+^ channels, and sarcoplasmic reticulum (SR) Ca^2+^-ATPase (SERCA). Recent data imply a novel role of Ca^2+^-influx via Ca_V_3.2 T-type channels to trigger Ca^2+^-sparks [5,6], contributing to ~25% of all Ca^2+^-sparks triggered at physiological membrane potentials in mesenteric arteries. Ca_v_3.2 apparently activate RyR2s from the cytosolic side [7], possibly due to the low unitary conductance of T-type channels, therefore the triggering comprises low amounts of total Ca^2+^ transferred into the cells and a more restricted spatial range than Ca^2+^-sparks triggered by L-type Ca_V_1.2 channels. Consistent with this mechanism, proper function of the T-type Ca_V_3.2-RyR axis requires sufficiently high SR Ca^2+^ load, which is regulated *via* Ca^2+^-influx through L-type Ca_V_1.2 channels [7].

There is a large body of experimental knowledge on key control processes of VSMC contractile state. However, the studies were usually performed in young adult rodents, whereas hypertension is a typical cardiovascular risk factor in humans at advanced age. We therefore extended our studies to aged mice, using approaches of pharmacological blockade (L-type channels: Cd^2+^, T-type channels: Ni^2+^, TRP channels: Gd^3+^) and specific knockout mice (Ca_V_1.2-KO/SMAKO, Ca_V_3.2-KO, EHD2-KO, Caveolin-1-KO). We found that Ca_V_1.2 L-type channels contribute to the same extent to Ca^2+^-sparks generation in young and aged mice. RyR2 is the predominant RyR isoform responsible for Ca^2+^-sparks [3], probably not only for Ca_V_1.2-mediated Ca^2+^-sparks but also for additional pathways. We found that the Ca_V_1.2-RyR2 axis works efficiently in old VSMCs, i.e increasing age does not impair the formation of Ca^2+^-sparks in old VSMCs [7].

Although Ca_V_3.2-deficiency (KO) does not affect blood pressure in young mice [8], Ca_v_3.2 channels limit myogenic tone in young, but surprisingly not in old mice mesenteric arteries [3,7,8]. Decreased Ca_V_3.2 channel expression/activity to trigger Ca^2+^ sparks are possible underlying mechanisms for these effects [7]. The newly discovered Ca_V_3.2-T-type channel Ca^2+^-influx pathway obviously exists only in young mice, but not in mice at advanced age.

Advanced age can alter the composition of lipid rafts and caveolae, which could affect a variety of signaling molecules contributing to the pathophysiology of cardiovascular diseases. Since localization of Ca_V_3.2 in caveolae close to RyRs is essential for triggering Ca^2+^-sparks, we analysed age-related alterations in caveolae to explain the complete malfunction of the Ca_v_3.2-RyR2 axis in old mice VSMCs. Treatment with methyl-ß-cyclodextrin, depleting cholesterol as an essential caveolae component from the plasma membrane, failed to inhibit Ca^2+^-sparks in VSMCs of old mice. Electron microscopy images revealed a decrease in caveolae density and alterations in caveolae structure in advanced age [7]. Thus, defective caveolae-RyR2 coupling might be caused by age-related ultrastructural alterations of caveolae and impaired Ca_V_3.2-Ca^2+^ sparks signaling by putative caveolemmal T-type Ca_V_3.2 channels, which are insufficiently close to RyRs for extracellular Ca^2+^-influx through T-type channels to trigger Ca^2+^-sparks. Of note, we confirmed our findings using novel Eps15 homology domain-containing protein (EHD2)-deficient mice with genetically impaired caveolae formation. In this animal model, Ca_V_3.2-expression was also decreased. Together, reduced caveolae density could downregulate Ca_V_3.2 T-type channels to impair the ability of T-type Ca_V_3.2 to generate Ca^2+^-sparks in old VSMCs.

In mice at advanced age, there was a fraction of Ca^2+^-sparks remaining after L-type channel block. This finding enabled us to study effects of the non-specific TRP channel blocker Gd^3+^ (100 µM) to shed a light on the putative role of an TRP channel(s) in VSM elementary Ca^2+^ release (Ca^2+^-sparks) to compensate for loss of Ca_V_3.2 T-type channels. We found that caveolae are presumably not required for the gadolinium-sensitive Ca^2+^-influx pathway, since caveolae disruption by methyl-ß-cyclodextrin did not alter Ca^2+^-spark events in old VSMCs. Due to the low specificity of Gd^3+^, further work is required to ascertain which TRP cation channel(s) or pathways are responsible for generation of these Ca^2+^ sparks.

**Figure 1:**
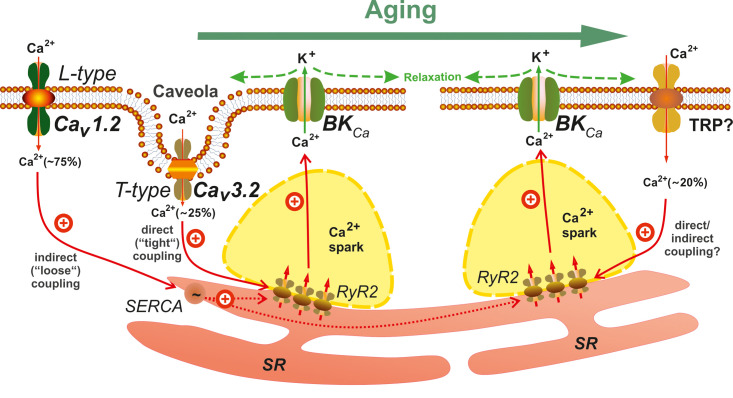
**Main Ca^2+^ influx pathways regulating Ca^2+^-sparks in young and aged mesenteric vascular smooth muscle cells (VSMCs).** Opening of clustered RyRs in the SR produces Ca^2+^-sparks that activate BK_Ca_ channels providing a negative feedback effect on vasoconstriction. Ca_V_1.2 L-type channels contribute to global cytosolic [Ca^2+^], thereby influencing luminal SR calcium (via SERCA) and generating the majority (75%) of Ca^2+^-sparks. Caveolae position Ca_V_3.2 T-type channels close to RyRs for extracellular Ca^2+^-influx to trigger (~25%) Ca^2+^-sparks. In aged mice VSMCs, this Ca_V_3.2-RyR pathway is lost. Instead, a gadolinium-sensitive Ca^2+^-influx pathway triggering (20%) Ca^2+^-sparks is upregulated. Nonselective TRP channels might be involved in this pathway. BK_Ca_, Ca^2+^-activated K^+^ channels; RyR2, ryanodine receptor subtype 2; SERCA, sarcoplasmic/endoplasmic calcium pump; SR, sarcoplasmic reticulum.

Our results revealed age-dependent differences in elementary Ca^2+^ signaling and myogenic tone regulation. The Ca_V_3.2-RyR-axis is an effective pathway to generate Ca^2+^-sparks in young VSMCs, but plays little or no role in aged VSMCs. A Gd^3+^-sensitive Ca^2+^-influx pathway, putatively through nonselective TRP channels, compensates for loss of the Ca_V_3.2-RyR-axis. Clarifying the exact identity of the receptor(s)/pathways and the nature of tight vs. loose coupling between Ca^2+^ influx-RyR2s pathways (including possible further participating proteins, e. g. SERCA, IP_3_Rs) will reveal potential novel targets for antihypertensive therapies in patients in the elderly (Figure 1).
